# An unusual case of congenital melanocytic nevus presenting as neurocutaneous melanoma coexisting with Tuberous Sclerosis complex: A case report

**DOI:** 10.1186/1752-1947-5-267

**Published:** 2011-07-01

**Authors:** Santosh Rai, Piyush Kalakoti, MM Aarif Syed, Purujit J Thacker, Rishi Jain, Gaurav Kalra

**Affiliations:** 1 Department of Surgery, Rural Medical College, Loni, Maharashtra, 413736, India; 2 Pravara Rural Hospital and Rural Medical College, Loni, Maharashtra, 413736, India

## Abstract

**Introduction:**

Congenital melanocytic nevi are among the several known risk factors for the development of melanoma. Neurocutaneous melanosis is a rare, congenital, non-hereditary disorder characterized by the presence of multiple and/or giant congenital melanocytic nevi. It is a rare condition, with fewer than 200 cases reported in the literature. Its association with tuberous sclerosis complex, a form of the neurocutaneous syndrome, is an unusual finding which, to the best of our knowledge, has not been documented in the English literature so far. Herein we present the first case documenting such an association in a 16-year-old post-pubertal Indian girl.

**Case presentation:**

In this report, we describe the case of a 16-year-old Indian girl who presented to our hospital with swelling on the scalp which had progressed from the hairline to just above the left brow, causing mechanical ptosis. She was born with a black-pigmented triangular patch covered with hair over the scalp which had increased in size over a period of eight years after birth. An X-ray of her skull and ultrasonography revealed soft tissue swelling in the left temporofrontoparietal region. Magnetic resonance imaging of her brain showed the presence of 8.99 cm × 2.26 cm abnormal signal intensity involving the scalp, a few small tubers with cortical dysplasia in the left frontoparietal region with asymmetric dilatation, and the presence of calcified subependymal nodules within the left lateral ventricle. These findings were suggestive of tuberous sclerosis. A histopathological examination of the swelling was suggestive of congenital melanocytic nevi. The patient underwent surgery. Excision of the tumor with primary skin grafting was done, with the graft being taken from the medial aspect of the right thigh.

**Conclusion:**

This case warrants further research to provide concrete evidence of an association of neurocutaneous melanoma with tuberous sclerosis complex. Research should be conducted to prove whether this is an unusual association or a new syndrome. Also, similar cases in other parts of the globe should be documented, as they would provide substantial support for such an association.

## Introduction

Congenital melanocytic nevi (CMN) are found in approximately 1% of newborn infants, but 90% of these nevi are very small. Giant congenital nevi (GCN), giant hairy nevi and nevocellular nevi represent a special group of melanocytic lesions that generally cover large areas of the body and carry a potential risk for the development of malignant melanoma [1]. A congenital nevus is one of several known risk factors leading to the development of melanoma. Fortunately, melanoma remains an uncommon malignancy in pre-pubertal children, with an annual incidence of 0.7 cases per 1 million children from birth through age nine years. Patients' concern regarding changing or worrisome-looking nevi are nonetheless very common. Moreover, by the time a child reaches adolescence, the incidence of melanoma increases substantially, with a rate of 13.2 cases per one million children ages 15 to 19 years [2].

Neurocutaneous melanosis (NCM) is a congenital, non-hereditary disorder defined by the presence of multiple and/or GCMN associated with abnormal melanin deposits in the brain and/or leptomeninges documented by magnetic resonance imaging (MRI) or autopsy [3-6]. It is a rare condition, with fewer than 200 cases reported in the literature. Although there is proliferation of melanocytes in the skin and arachnoid matter, there is currently no embryological explanation for the genesis of this disorder. Approximately one-half of all patients with NCM eventually develop melanoma of the central nervous system [7]. However, the presence of tuberous sclerosis complex (TSC), a form of the neurocutaneous syndrome (NCS), concurrently with NCM is an unusual and rare finding which, to the best of our knowledge, has not been documented in the literature so far. Herein we present the first such association in a 16-year-old girl.

## Case presentation

A 16-year-old Indian girl presented to our hospital with swelling on the scalp. She was born with a black-pigmented triangular patch over the scalp covered with hair as well as multiple black hairy patches on her extremities, back, and most of her anterior trunk. The patch on the scalp had increased in size over a period of eight years after birth. Within the past three months, swelling had progressed from the hairline to just above the left brow, leading to unilateral mechanical ptosis. Upon applying pressure, there was a scanty, yellowish, non-foul-smelling discharge that was occasionally blood-stained and was associated with intense itching without any pain. Her sleep, appetite, bowel, and bladder were unaltered. Her menarche had begun at the age of 15 years, and her menstrual cycle was normal and regular. Her birth and developmental history were normal. The family history was negative for congenital nevi or melanoma.

The patient's clinical examination revealed her to be afebrile, with a pulse rate of 64 beats/minute, a respiratory rate of 16 breaths/minute, and blood pressure of 120/70 mmHg. The examination of her cardiovascular system was normal. Her cranial nerve functions were normal, and no neurological deficit was found in the limbs. The plantar response in both the limbs was flexor. There was no evidence of meningeal irritation.

**Figure 1:**
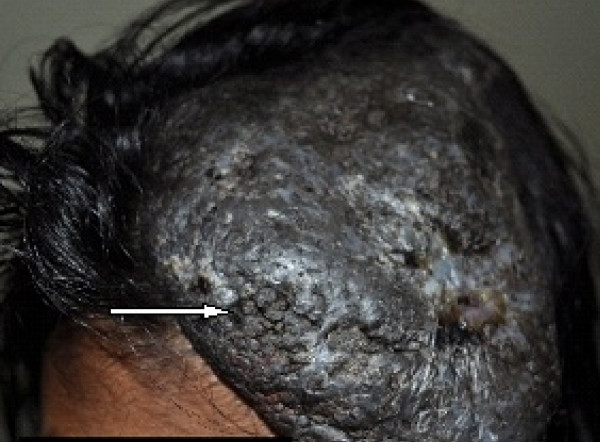
**Gross appearance of the lesion**. A single swelling of size 10 cm × 8 cm (white arrow) on the left side of the scalp extending anteroposteriorly from 3 cm in front of the left parietal eminence to just above the lateral part of the left supraorbital ridge is shown. The transverse extension from the line passing through the left parietal eminence to the right of the midline can be seen.

A local examination revealed a single swelling of 10 cm × 8 cm on the left side of the scalp. The swelling extended anteroposteriorly from 3 cm in front of the left parietal eminence to just above the lateral part of the left supraorbital ridge. The transverse extension was from the line passing through the left parietal eminence to the right of the midline (Figure 1). Her local temperature was not raised, and tenderness was absent. The surface was irregular, rough with multiple pits, and the margins were irregular with rounded edges. The swelling was firm in consistency and mobile. The anterior part of the swelling overlying the forehead could be lifted. The underlying skin appeared normal. It was non-pulsatile with no evidence of impulse on coughing. The regional lymph nodes were not palpable. Numerous hairy nevi were present over the scalp, trunk, and limbs.

The patient was referred to the Department of Neuro-ophthalmology and Otolaryngology for further assessment. Her fundoscopy and visual evoked potentials were normal. Her otorhinolaryngological evaluations were normal.

Routine blood investigations showed that her hemoglobin level was 11.6% and her total leukocyte count was 4200/mm^3 ^with relative neutrophilia (80%). Her serum urea and electrolyte levels were normal. A lumbar puncture revealed clear cerebrospinal fluid with a normal cell count and biochemistry and no growth on culture. Her liver and kidney function tests were within normal limits. Her electrocardiogram did not show any abnormal features.

An X-ray of her skull (posteroanterior view) revealed a soft tissue swelling in the left temporofrontoparietal region. Ultrasonography of the swelling showed a large, homogeneous, hypoechoic, solid 9.4 cm × 6.8 cm × 1 cm lesion in her scalp in the left frontal region and partially extending into the parietal region on the left side. The underlying outer table of the skull and diploic spaces were normal with few hyper-reflective areas scattered throughout the lesion and distal shadowing raising clinical suspicions of tiny calcified foci. The interface between the lesion and the scalp was obscured. No significant color flow was noted on a color Doppler ultrasonogram. These findings were highly suggestive of a possible angiofibroma. Power Doppler ultrasonography showed a highly vascularized lesion.

**Figure 2:**
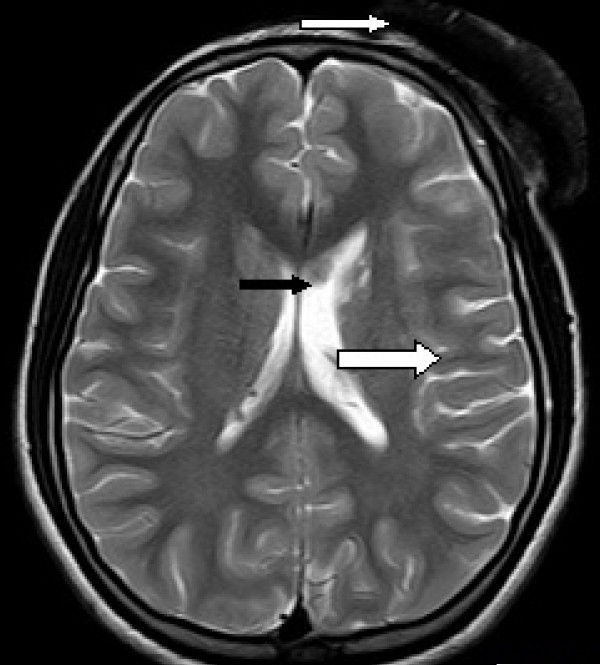
**Magnetic resonance imaging findings**. These MRI scans show a single 8.99 cm × 2.26 cm abnormal signal intensity involving the scalp in the left frontoparietal region appearing isointense to hyperintense on T1-weighted images (thin white arrow) with few calcified subependymal nodules within the body of the lateral ventricles on the right side (black arrow), a few small tubers with cortical dysplasia (thick white arrow) in the left frontoparietal region, and left maxillary sinusitis.

MRI of her brain (both plain and contrast images) was done by using a multi-echo, multi-planar technique, which showed the presence of a 8.99 cm × 2.26 cm abnormal signal intensity involving the scalp (subcutaneous plane within fat) in the left frontoparietal region. The signal intensity appeared isointense to hyperintense on T1-weighted MRI scans with few small tubers with cortical dysplasia in the left frontoparietal region and asymmetric dilatation of the left ventricle that were hypointense on T2-weighted MRI and fluid attenuated inversion recovery (FLAIR) images. Few foci were seen within the lesion, which appeared hyperintense on T2-weighted MRI and FLAIR images. The MRI impression revealed multiple intra-cranial lesions, which included a benign, homogeneously enhancing, subcutaneous scalp lesion in left frontoparietal region suggestive of angiofibroma; a few calcified subependymal nodules within the body of the lateral ventricles on the right side; a few small tubers with cortical dysplasia in the left frontoparietal region; and left maxillary sinusitis (Figure 2). These radiological findings were highly suggestive of tuberous sclerosis. Magnetic resonance angiography revealed the absence of involvement of any underlying blood vessels.

**Figure 3:**
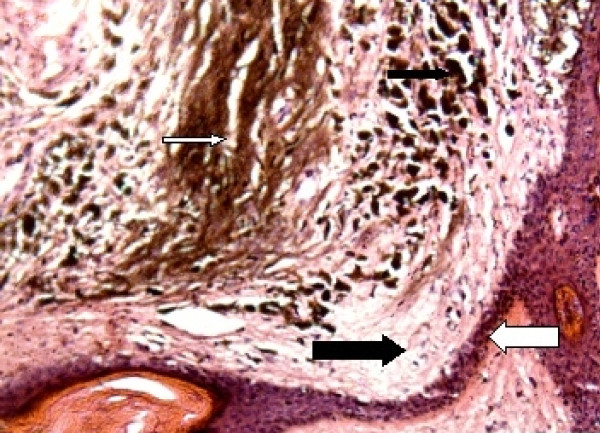
**Pathologic findings (hematoxylin and eosin stain)**. This high-power photomicrograph of the area shows the keratinized, stratified squamous epithelium (thick black arrow) and the epidermodermal junction (thick white arrow). The epidermis is thinned out with loss of rete pegs. The dermis shows diffuse deposition of melanin pigment (thin white arrow) and the presence of melanocytes (thin black arrow) around and within the hair follicles.

Microscopic examination of the patient's scalp swelling revealed the presence of lining keratinized, stratified squamous epithelium and underlying dermis. The epidermis was thinned out with loss of rete pegs. Her dermis showed lobules and nests of nevi cells, hair follicles, sweat glands, and sebaceous glands. There was diffuse deposition of melanin pigment and the presence of melanocytes around and within the hair follicles and sebaceous glands extending up to deep subcutis and infiltrating the fat (Figure 3). All of these findings were suggestive of congenital melanocytic nevi.

**Figure 4:**
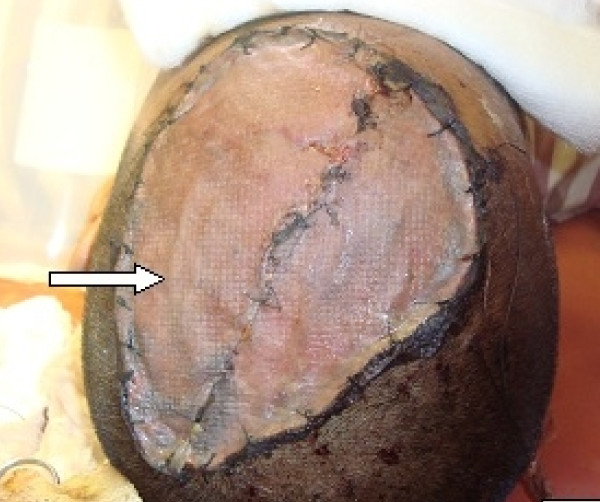
**Post-operative photograph of the scalp**. A primary skin graft was placed over the affected area (white arrow) after removal of the tumor.

On correlating these imaging findings with the patient's clinical symptoms, surgery was considered. Excision with primary skin grafting was done (Figure 4). The graft was taken from the medial aspect of her right thigh.

## Discussion

In 1861, Rokitansky [8] first described NCM in a 14-year-old girl. CNM is a well-known risk factor for the eventual development of melanoma. CNM are classified as large (≥ 20 cm), medium (1.5 cm to 19.9 cm), and small (≤ 1.5 cm) nevi according to their size [9]. The term "multiple" is used when more than three lesions are present. NCM is thought to be a result of an error occurring during morphogenesis in the neuroectoderm [10]; however, its exact pathogenesis is unclear. NCM is currently diagnosed according to the following criteria put forth by Kadonaga and Frieden [10] in 1991: (1) large and/or multiple CMN in association with meningeal melanosis or melanoma, (2) no evidence of cutaneous melanoma, except in patients with histologically benign meningeal lesions, or (3) no evidence of meningeal melanoma, except in patients with histologically benign cutaneous lesions. They also found that 66% of NCM patients had large nevi, and the remaining 34% had numerous pigmented lesions in the absence of a single, large congenital melanocytic nevus. In their study, all NCM patients had either posterior midline nevi or head and neck lesions, which suggest that the axial distribution is an important risk factor for developing NCM. Our patient also had a single, large CMN with an axial distribution over the scalp with no evidence of meningeal melanoma, but she had histologically benign cutaneous scalp lesions, pointing toward the diagnosis of NCM.

Clinically, patients may present with a mass lesion or increased intra-cranial pressure due to hydrocephalus, cranial nerve paralysis, myelopathy, convulsive seizures, and so forth [11]. Most cases of melanoma arising within the GCN occur before puberty [12], with a reported incidence of 8.52% and a lifetime risk in the range of 2.3% [13-17]. Giant congenital melanocytic nevi (GMCN) occur in approximately one in 20,000 people, and about 100 cases were reported worldwide prior to 2000 [3,18,19]. It is recommended that GCMN be removed soon after the diagnosis because of cosmetic problems and their propensity for malignant change [10,18,20-23]. The neurological manifestations of NCM vary with age [10]. Before the age of two years, the most common initial clinical signs and symptoms of NCM are related to increased intra-cranial pressure, including headache (35%), vomiting (42%), generalized seizures (48%), increased head circumference (23%), cranial nerve palsies (26%; in particular cranial nerve VI), papilledema (10%), and meningeal signs (3%) [24]. The subset of patients with a discrete intra-cranial mass become symptomatic when they are older (mean age, 12.8 years; age range, birth to 65 years) and are more likely to develop focal seizures, localized sensorimotor deficits, difficulties with speech, or psychiatric symptoms [24]. The prognosis for patients with symptomatic NCM is poor. Our patient was asymptomatic at the time of presentation and presented to our hospital only for aesthetic reasons.

The present case pointed toward a definite diagnosis of tuberous sclerosis complex because of two major features: calcified subependymal nodules within the body of the lateral ventricles and cortical tubers as confirmed by MRI. There was no history suggestive of TSC in her parents or siblings.

Our patient had congenital giant, hairy melanotic nevi of the skin (scalp) which had gradually progressed in size to a cutaneous scalp tumor indicative of melanoma. NCM is known to be associated with the other NCSs, such as Sturge-Weber syndrome and von Recklinghausen's disease. Associations with Dandy-Walker complex, spinal lipomas, and arachnoid cysts have also been reported [25,26]. About 100 cases of NCM have been reported. However, the coexistence of tuberous sclerosis with NCM is an unusual finding which, to the best of our knowledge, has not been documented in the literature so far.

## Conclusion

This case warrants further research to provide concrete evidence of an association of TSC with NCM. Research should be conducted to prove whether NCM associated with TSC is an unusual or new syndrome. Also, similar cases occurring in other parts of the globe should be documented, as they would provide substantial support for such an association. Genetic and molecular investigations with specific tumor markers should be conducted. However, in the present case, because of the paucity of facilities in the institution and the financial constraints on the patient, a detailed investigation could not be performed.

## Consent

Written informed consent was obtained from the patient and the patient's next-of-kin for publication of this case report and any accompanying images. A copy of the written consent is available for review by the Editor-in-Chief of this journal.

## Competing interests

The authors declare that they have no competing interests.

## Authors' contributions

SR, PK, and MMAS participated in the clinical diagnosis, sequence alignment, and drafting of the manuscript and made useful contributions to the review of the literature. GK, RJ, PK, and MMAS were the team of operating surgeons. PK and MMAS participated in writing the Discussion section. PK and MMAS and PJT helped in the revision of the manuscript. All authors read and approved the final manuscript.
